# CHPF promotes gastric cancer tumorigenesis through the activation of E2F1

**DOI:** 10.1038/s41419-021-04148-y

**Published:** 2021-09-25

**Authors:** Xiaolin Lin, Ting Han, Qing Xia, Jiujie Cui, Meng Zhuo, Yiyi Liang, Wenyu Su, Lisha Wang, Liwei Wang, Zebing Liu, Xiuying Xiao

**Affiliations:** 1 Department of Oncology, Ren Ji Hospital, Shanghai Jiao Tong University School of Medicine, Shanghai, 200127 China; 2 Department of Gastroenterology, Ren Ji Hospital, Shanghai Jiao Tong University School of Medicine, Shanghai Institute of Digestive Disease, Shanghai, 200127 China; 3 Michigan Center for Translational Pathology, University of Michigan Medical School, Ann Arbor, 48109 MI USA; 4 Department of Pathology, Ren Ji Hospital, Shanghai Jiao Tong University School of Medicine, Shanghai, 200127 China

**Keywords:** Gastric cancer, Cell biology

## Abstract

Chondroitin polymerizing factor (CHPF) is an important glycosyltransferase involved in the biosynthesis of chondroitin sulfate. However, the relationship between CHPF and gastric cancer has not been fully investigated. CHPF expression in gastric cancer tissues was detected by immunohistochemistry and correlated with gastric cancer patient prognosis. Cultured gastric cancer cells and human gastric epithelial cell line GES1 were used to investigate the effects of shCHPF and shE2F1 on the development and progression of gastric cancer by MTT, western blotting, flow cytometry analysis of cell apoptosis, colony formation, transwell and gastric cancer xenograft mouse models, in vitro and in vivo. In gastric cancer tissues, CHPF was found to be significantly upregulated, and its expression correlated with tumor infiltration and advanced tumor stage and shorter patient survival in gastric cancer. CHPF may promote gastric cancer development by regulating cell proliferation, colony formation, cell apoptosis and cell migration, while knockdown induced the opposite effects. Moreover, the results from in vivo experiments demonstrated that tumor growth was suppressed by CHPF knockdown. Additionally, E2F1 was identified as a potential downstream target of CHPF in the regulation of gastric cancer, and its knockdown decreased the CHPF-induced promotion of gastric cancer. Mechanistic study revealed that CHPF may regulate E2F1 through affecting UBE2T-mediated E2F1 ubiquitination. This study showed, for the first time, that CHPF is a potential prognostic indicator and tumor promoter in gastric cancer whose function is likely carried out through the regulation of E2F1.

## Introduction

Gastric cancer (GC), one of the most commonly diagnosed malignant tumors, has the fifth highest incidence rate and the third highest mortality rate among all cancer types [1, 2]. To date, comprehensive therapy based on surgery is the most effective strategy for the treatment of gastric cancer [3, 4]. However, due to the atypical early symptoms of gastric cancer, most gastric cancer patients are diagnosed with advanced-stage tumors, which decreases the chance of resection and results in a poor 5-year survival rate [5]. The occurrence and development of gastric cancer is a biological process consisting of multiple stages, including the activation of oncogenes and the inactivation of tumor suppressor genes [6]. Although the therapeutic effect in gastric cancer has improved with the emergence of targeted drugs developed based on molecular biology research of gastric cancer [7], the 5-year survival rate of gastric cancer has not improved satisfactorily [4]. Therefore, there is an urgent need to further explore the molecular mechanism of gastric cancer to develop novel biomarkers and therapeutic targets, which will help to overcome the limitations associated with the diagnosis and treatment of gastric cancer.

Chondroitin sulfate (CS) is a type of linear polysaccharide that is composed of repeated disaccharide residues and modified by sulfated residues at multiple sites [8]. Due in part to its structural diversity caused by chain length and sulfation sites, chondroitin sulfate plays an important role in many cellular biological functions, such as cell adhesion, cell differentiation and neural network formation [9, 10]. Chondroitin polymerizing factor (CHPF), one of the six key glycosyltransferases in the biosynthesis of chondroitin sulfate, is located in the 2q35-q36 region of the human chromosome, spanning four exon regions, and plays an important role in cell function [11]. More importantly, CHPF is abnormally upregulated in several types of cancer and has been proven to be a potential tumor promoter in colorectal cancer [12], head and neck squamous cell carcinoma [13], and glioma [14]. However, the role of CHPF in gastric cancer has not been reported until now and remains largely unknown.

Therefore, our current study is the first to reveal the expression pattern and functions of CHPF in gastric cancer. The results demonstrated that CHPF was upregulated in gastric cancer tissues compared with normal tissues, and a high CHPF expression level was correlated with Tumor cell infiltration, advanced tumor stage, and poor prognosis. Moreover, further investigation revealed that CHPF could promote proliferation and migration, and inhibit apoptosis of gastric cancer cells through the regulation of E2F1. Therefore, these findings suggest a role for CHPF in promoting the development and progression of gastric cancer and indicate that CHPF may serve as a prognostic indicator of gastric cancer and a therapeutic target in gastric cancer treatment.

## Materials and methods

### Cell culture and lentivirus infection

The gastric carcinoma cell lines used in the present study were purchased from BeNa Technology (Hangzhou, Zhejiang, China). The detailed information on the cell lines and the specific culture conditions are listed below. AGS cells were cultured in 90% F-12 medium with 10% FBS (Gibco, Rockville, MD, USA), while SGC-7901 and BGC-823 cells were maintained in RPMI-1640 (Gibco, Rockville, MD, USA) medium containing 10% FBS at 37 °C with 5% CO_2_. MGC-803 cells were cultured in 90% DMEM (Gibco, Rockville, MD, USA) containing 10% FBS. The medium was changed every 3 or 4 days.

AGS and SGC-7901 cells in the logarithmic growth phase were used for lentivirus infection. After the cells were harvested and washed with PBS, cells at a density of 3 × 10^6^ cells/mL were seeded in culture medium containing 10% FBS, after which lentivirus and control vectors were added, and the cells were incubated in a 5% CO_2_ incubator at 37 °C. After 72 h, the infection efficiency (>80% was considered a successful infection) was observed under a fluorescence microscope.

Cycloheximide (CHX, an inhibitor of protein synthesis) was purchased from Selleck (Cat. S7418) and used at a concentration of 0.2 mg/mL. Z-Leu-Leu-Leu-al (MG132, an inhibitor of the ubiquitin–proteasome pathway) was purchased from MEC (Cat. HY-13259) and used at a concentration of 20 μM.

### Immunohistochemistry analysis

The formalin-fixed tissue microarray (TMA) of gastric cancer and paired normal tissues from 140 patients were obtained from Renji Hospital (Shanghai, China). The age of patients ranged from 20 to 40 years, and other related information was also collected. Written informed consent was collected from all patients. Our study was approved by the Ethics Committee of Renji Hospital, Shanghai Jiao Tong University School of Medicine.

First, the TMA was dewaxed, hydrated and washed; thereafter, the TMA was subjected to antigen retrieval with citric acid buffer by heating at 120 °C. Then, the TMA was blocked with H_2_O_2_ at a concentration of 3% and subsequently incubated with anti-CHPF (1:200) or anti-E2F1 (1:100) overnight at 4 °C, followed by incubation with HRP-conjugated secondary antibody for 2 h at room temperature. Next, the TMA was stained by DAB and hematoxylin. CHPF and E2F1 expression was observed with ImageScope and CaseViewer and then quantified for analysis using IHC scores. The staining extent was graded as 0 (0%), 1 (1–25%), 2 (26–50%), 3 (51–75%), or 4 (76–100%). The staining intensity varied from weak to strong. Specimens were classified into negative (0), positive (1–4), ++ positive (5–8), or +++ positive (9–12) based on the sum of the staining intensity and staining extent scores. The antibodies used in IHC are listed in Table S2.

### Establishment of shCHPF and shE2F1 cells

To silence CHPF and E2F1, shRNA, lentivirus, and related vector control were constructed by Shanghai Biosciences Co., Ltd. (Shanghai, China). The target sequence of CHPF designed in this study was 5′-AGCTGGCCATGCTACTCTTTG-3′ and those of E2F1 were as follows: 5′-GGGCATCCAGCTCATTGCCAA-3′ (10794), 5′-CAGCTGGACCACCTGATGAAT-3′ (10795), and 5′-GACCTCTTCGACTGTGACTTT-3′ (10796). Moreover, full-length CHPF was cloned into the lentivirus vector Ubi-MCS-3FLAG-CBh-gcGFP-IRES-puromycin (BR-V112) (Shanghai Biosciences, Shanghai, China) to construct CHPF-overexpressing cells. Transfection of shRNA was performed using Lipofectamine 2000 Reagent (Thermo Scientific Oxoid™, Waltham, MA, USA). Puromycin (Takara Bio, Otsu, Japan) was utilized to screen the transfected cells, which were further verified by observing GFP fluorescence by a fluorescence microscope (Olympus, Tokyo, Japan).

### Quantitative RT-PCR

Total RNA from SGC-7901 and AGS cells was isolated using TRIzol reagent (Thermo Fisher Scientific, Waltham, MA, USA) according to the manufacturer’s instructions. The purity and concentration of the RNA were assessed by a NanoDrop 2000 spectrophotometer (Thermo Fisher Scientific, Waltham, MA, USA), and 1.0 μg of total RNA was reverse transcribed to obtain high-quality cDNA using M-MLV Reverse Transcriptase (Thermo Fisher Scientific, Waltham, MA, USA) according to the manufacturer’s protocol. The qPCR system was 10 μL and was performed using AceQ qPCR SYBR Green Master Mix (Vazyme, Nangjing, Jiangsu, China). The PCR cycling conditions were as follows: predenaturation at 95 °C for 30 s and 40 cycles of denaturation at 95 °C for 10 s, annealing at 60 °C for 30 s, and extension at 72 °C for 30 s. Gene expression quantification was performed using the 2^−∆∆Ct^ method. GAPDH served as an endogenous control. The primer sequences used in PCR are listed in Table S1.

### Western blotting (WB) and co-immunoprecipitation (co-IP)

Total proteins from each group of cells were extracted using ice-cold RIPA lysis buffer, and the concentration of proteins was determined by a BCA protein reagent kit (HyClone-Pierce, Logan, UT, USA). A total of 20 μg of protein was separated by 10% SDS-PAGE gels and transferred to PVDF membranes. The membrane was blocked with 5% BSA for 1 h and was then incubated with primary antibodies at 4 °C overnight, followed by HRP-labeled goat anti-rabbit secondary antibody. An Immobilon Western Chemiluminescent HRP Substrate (ECL-Plus™) kit (Millipore, Schwalbach, Germany) was used for visualization, and proteins were detected with an X-ray imaging analyzer (Kodak, Rochester, NY, USA). GAPDH was used as the internal standard. Each test was performed on the same membrane and repeated 3 times. For co-IP assay, total protein obtained from corresponding cells was used for immunoprecipitation with anti-E2F1 or anti-UBE2T, followed by the detection of protein expression by western blot with indicated antibodies. All antibodies used in WB are listed in Table S2.

### Celigo cell counting assay

Logarithmic growth phase lentivirus-infected SGC-7901 cells in the groups CHPF, shCtrl, shE2F1, NC (KD + OE), and CHPF + shE2F1 were harvested and trypsinized. Then, all cells were resuspended in 90% 1640 medium (10% FBS) to achieve a cell density of 2 × 10^5^ cells/mL. A 100 μL cell suspension was seeded in each well of a 96-well plate and cultured for 5 days. Each group included three wells. The plate was continuously detected by Celigo (Nexcelom, Lawrence, MA, USA) for 5 days, and the proliferation rate was analyzed once a day at the same time.

### MTT assay

AGS and SGC-7901 cells in the exponential growth phase were trypsinized and then seeded into a 96-well plate (2000 cells/well). After that, the cells were incubated for 4 h after adding 20 μL of MTT solution (5 mg/mL, GenView, El Monte, CA, USA) followed by 100 μL of DMSO solution. The absorbance values at 490 nm were measured by a microplate reader (Tecan, Männedorf, Zürich, Switzerland), and the reference wavelength was 570 nm. The cell viability ratio was calculated according to the equation cell viability (%) = optical density (OD) treated/OD control × 100%.

### Flow cytometry analysis of cell apoptosis

An Annexin-V APC Apoptosis Detection Kit (eBioscience, San Diego, CA, USA) was used for cell apoptosis detection with flow cytometry according to the manufacturer’s instructions. Lentivirus-infected AGS and SGC-7901 cells were collected, washed, trypsin digested, and seeded in a 6-well plate with 2 mL medium. Finally, the cells were stained in the dark by using Annexin-V APC buffer, and flow cytometry was used to detect the cell status. The cell apoptosis rate was analyzed.

### Colony formation assay

Lentivirus-infected AGS and SGC-7901 cells were cultured for 5 days and then collected, digested and resuspended. When the cell density was adjusted to 400 cells/mL, a 2 mL cell suspension was seeded in a 6-well plate and cultured for 10 days to form colonies, and the medium was changed every 3 days during this process. Imaging was performed with a fluorescence microscope (Olympus, Tokyo, Japan). Then, the cells were fixed with 4% paraformaldehyde and stained with Giemsa (Dingguo, Shanghai, China), and the number of colonies (>50 cells/colony) was counted.

### Wound-healing assay

Lentivirus-infected AGS and SGC-7901 cells were seeded in a 96-well plate and cultured until the cell confluence reached 90%. A replicator with 96 pins (VP scientific, San Diego, CA, USA) was used to generate scratches, and the plate was washed 3 times with PBS. Then, the cells were cultured with serum-free medium for indicated time. Images were collected at 24 and 48 h, and the migration rate was analyzed by Cellomics (Thermo, Waltham, MA, USA).

### Transwell assay

Transwell chambers for 24-well plates (Corning, NY, USA) were prepared in advance, and 100 µL medium without FBS was added to the upper chamber with 5 × 10^4^ cells. Next, 600 µL medium with 30% FBS, serving as the chemoattractant, was added into the lower chamber. Cells were cultured for 44 h, and then the transmigrated cells were fixed with 4% paraformaldehyde and stained using crystal violet after removing the non-transmigrated cells. Fluorescence microscopy (Olympus, Tokyo, Japan) was used for imaging and cell counting.

### Human apoptosis antibody array

The apoptosis signaling pathway was examined using the Human Apoptosis Antibody Array Kit (Abcam, Cambridge, MA, USA) according to the manufacturer’s protocol. The levels of signaling pathway-related proteins were visualized using a ChemiDoc XRS chemiluminescence detection and imaging system using an HRP-conjugated secondary antibody, and each array membrane was exposed to X-ray film. The density of the spots was quantitated using Quantity One software. We repeated the same experiment 3 times.

### RNA sequencing

RNA sequencing analysis was performed by Shanghai Biosciences Co., Ltd. (Shanghai, China). In brief, RNA from shCtrl and shCHPF SGC-7901 cells was extracted by an RNeasy kit (Sigma-Aldrich, St. Louis, MO, USA) according to the manufacturer’s protocol. The quality of total RNA was valued using a NanoDrop 2000 (Thermo Fisher Scientific, Waltham, MA, USA) and an Agilent 2100 Bioanalyzer (Agilent, Santa Clara, CA, USA). Then, RNA sequencing was performed by a 3′IVT Plus kit (Affymetrix, Santa Clara, CA, USA) according to the manufacturer’s instructions, and the outcomes were scanned using an Affymetrix Scanner 3000 (Affymetrix, Santa Clara, CA, USA). The statistical analysis of raw data was accomplished by using a Welch *t*-test with Benjamini-Hochberg FDR and applying a significant level of FDR < 0.05. The functional annotation enrichment analysis was performed with IPA (Qiagen, Hilden, Germany). The absolute value of the *Z* score >2 was considered meaningful.

### Mouse xenograft model

Four-week-old healthy female BALB/c nude mice (*n* = 20) were purchased from Shanghai SLAC Laboratory Animal Co., Ltd. (Shanghai, China) and were randomly divided into the shCtrl group and shCHPF group. Mouse xenograft models were constructed by subcutaneous injection of 4 × 10^6^ SGC-7901-shCtrl and SGC-7901-shCHPF cells. Data on growth markers, such as mouse weight, tumor length (*L*) and width (*W*), were collected 1–2 times per week (tumor volume = π/6 × *L* × *W* × *W*). In vivo imaging (Perkin Elmer, Waltham, MA, USA) was used to observe fluorescence before the mice were sacrificed. After sacrificing all mice by injecting 2% pentobarbital sodium, tumors were removed for analysis and weighing. All animal experiments performed in our study were approved by the Ethics Committee of Renji Hospital, Shanghai Jiao Tong University School of Medicine.

### Ki-67 immunostaining

Tumors from mouse xenograft models were collected, fixed with formalin and embedded using paraffin. Then, 2 μm sections were obtained and incubated in xylene. After incubation, the sections were immersed in 100% ethanol for 10 min and then blocked with PBS-H_2_O_2_ with 0.1% Tween 20 for 10 min at room temperature. Ki-67 primary antibody (1:200, # ab16667, Abcam) was added to all slides and incubated at 4 °C overnight. HRP goat anti-rabbit IgG secondary antibody (1:400) was added and further incubated for 2 h at room temperature. Stained slides were examined with a microscope.

### Statistical analysis

Categorical variables in our study are expressed as percentages, and continuous variables are expressed as the mean ± SD (*n* ≥ 3). The chi-squared test was applied to evaluate the CHPF expression differences between tumor tissues and normal tissues, and Mann-Whitney *U* analysis and Spearman rank correlation analysis were used to evaluate the association between CHPF expression and the characteristics of gastric cancer patients. The significant differences between different groups were analyzed using Student’s *t*-test. All analyses were performed using SPSS 17.0 software (Chicago, IL, USA), and *P* < 0.05 was considered significant.

## Results

### CHPF was upregulated in gastric cancer and associated with a poor prognosis

**Fig. 1:**
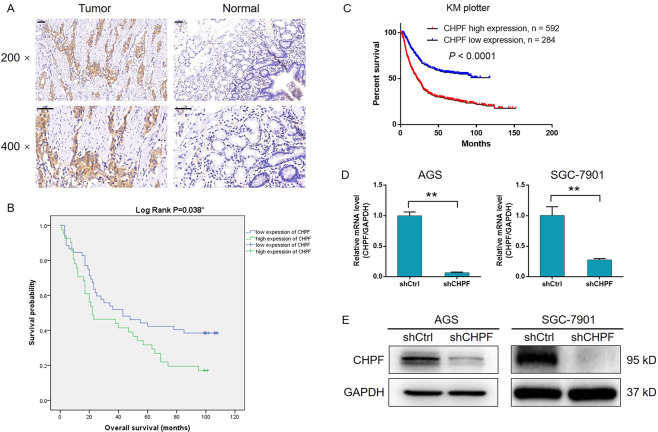
CHPF was upregulated in gastric cancer. **A** The expression of CHPF in gastric cancer tissues was detected by IHC analysis (scale bar = 50 μm). **B** The relationship between CHPF expression and the prognosis of gastric cancer patients. **C** The relationship between CHPF expression and the prognosis of gastric cancer patients was further analyzed by data mining of the KM plotter database. Cell models with CHPF knockdown were constructed through lentivirus infection, and the knockdown efficiency was verified by qPCR (**D**) and western blotting (**E**). The data are expressed as mean ± SD, ^**^P < 0.01.

CHPF expression in clinical specimens was detected by immunohistochemical analysis to explore the role of CHPF in gastric cancer. As indicated by representative images in Fig. 1A, a significant upregulation of CHPF expression in gastric cancer tissues compared with normal tissues was clearly observed. The expression level of CHPF in tumor tissues was also generally upregulated, as shown by the statistical analysis (Table 1, *P* < 0.001). Moreover, the highly abundant expression and upregulation of CHPF in gastric cancer cell lines compared with the human gastric epithelial cell line GES1 was also detected by qPCR (Fig. S1A). Analysis of the association between CHPF expression and the characteristics of gastric cancer patients was conducted to confirm the significance of the CHPF levels. The results showed that high CHPF expression was correlated with increased Tumor cell infiltration and advanced tumor stage (Table 2), which was further confirmed by Spearman rank correlation analysis (Table S3). Moreover, survival curves according to the Kaplan-Meier analysis indicated relatively poorer prognosis of patients with higher CHPF expression (log-rank *P* < 0.05, Fig. 1B), which was consistent with the data mining result of the gastric cancer database in KM plotter (Fig. 1C). Taken together, these results revealed the potential role of CHPF as a prognostic indicator and tumor promoter in the development of gastric cancer.

**Table 1:** Expression patterns in gastric cancer tissues and normal tissues revealed in immunohistochemistry analysis.

CHPF expression	Tumor tissue		Normal tissue		*P* value
	Cases	Percentage	Cases	Percentage	
Low	85	60.7%	118	99.2%	<0.001
High	55	39.3%	1	0.8%

**Table 2:** Relationship between CHPF expression and tumor characteristics in patients with gastric cancer.

Features	No. of patients	CHPF expression		*P* value
		Low	High	
All patients	140	85	55	
Age (years)				0.074
<58	68	47	21	
≥58	70	38	32	
Gender				0.619
Male	80	50	30	
Female	60	35	25	
Grade				0.333
1	1	1	0	
2	16	10	6	
3	110	68	42	
4	13	6	7	
T infiltrate				0.043
T1	19	17	2	
T2	10	6	4	
T3	64	36	28	
T4	46	25	21	
Lymphatic metastasis (N)				0.209
N0	42	29	13	
N1	22	14	8	
N2	32	17	15	
N3	42	24	18	
Stage				0.012
1	19	16	3	
2	41	28	13	
3	64	31	33	
4	15	9	6	
Tumor metastasis (M)				0.877
No	124	75	49	
Yes	16	10	6	
Tumor size				0.753
<5 cm	39	22	17	
≥5 cm	57	34	23	

### CHPF knockdown regulated the proliferation, migration, and apoptosis of gastric cancer cells

**Fig. 2:**
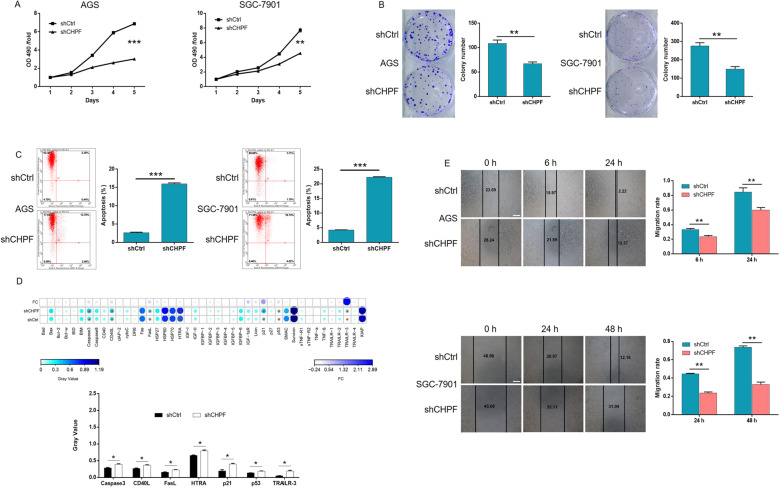
Knockdown of CHPF inhibited proliferation and migration and promoted apoptosis of gastric cancer cells. **A** The effects of CHPF knockdown on AGS and SGC-7901 cells were detected by MTT assay. **B** The results show the effects of CHPF on the colony formation ability of gastric cancer cells. **C** The effects of CHPF knockdown on gastric cell apoptosis were evaluated by flow cytometry. **D** The differentially expressed proteins in gastric cancer cells between the shCHPF and shCtrl groups were identified by a human apoptosis antibody microarray. **E** The effects of CHPF knockdown on the migration ability of gastric cancer cells were examined by wound-healing assay (scale bar = 200 μm). Representative images were selected from at least three independent experiments. The data are expressed as mean ± SD, ^*^P < 0.05, ^**^P < 0.01, ^***^P < 0.001.

Based on the above results, we subsequently examined the regulatory effects of CHPF knockdown on gastric cancer cells. Lentiviruses expressing shCHPF (designed for CHPF knockdown) or shCtrl (used as a negative control) were used to transfect the human gastric cancer cell lines AGS and SGC-7901. Fluorescence imaging based on the green fluorescent protein tag in the lentivirus vector proved a >80% transfection efficiency in both cell lines (Fig. S1B). Moreover, qPCR detection proved that CHPF expression in AGS and SGC-7901 cells was downregulated by more than 90% and 70%, respectively, which was consistent with the WB results (Fig. 1D, E). The established cell models were next subjected to the MTT assay to detect cell proliferation, which indicated significantly inhibited cell growth by CHPF knockdown, whereas the opposite results were obtained in the shCtrl group (> 40% inhibition, *P* < 0.01, Fig. 2A). Similarly, the colony formation ability of gastric cancer cells was found to be suppressed in the shCHPF group (> 40% inhibition, *P* < 0.01, Fig. 2B). Moreover, cell apoptosis was detected using both AGS and SGC-7901 cells, and a >7-fold increase in the apoptosis rate was demonstrated in the shCHPF group (*P* < 0.001, Fig. 2C). Through the human apoptosis antibody array, the mechanism by which CHPF regulated gastric cancer cell apoptosis was investigated, and the results revealed the involvement of Caspase-3, CD40L, FasL, HTRA, p21, p53, and TRAILR-3 (Fig. 2D). Furthermore, CHPF knockdown significantly inhibited the migration ability of gastric cancer cells (> 20% inhibition in AGS and >50% inhibition in SGC-7901 cells, *P* < 0.01, Fig. 2E). Overall, these results indicate that knockdown of CHPF may alleviate the development of gastric cancer by inhibiting cell proliferation, suppressing migration, and inducing apoptosis.

### Knockdown of CHPF inhibited the growth of gastric cancer in vivo

**Fig. 3:**
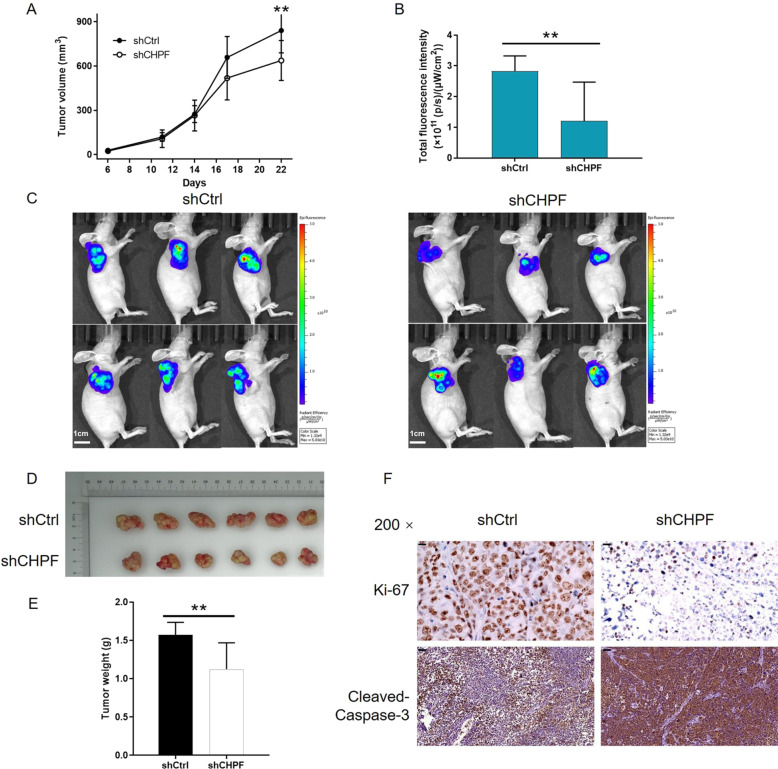
Knockdown of CHPF inhibited tumor growth in vivo. **A** The results showed that the tumor volume of the shCHPF group was smaller than that of the shCtrl group. **B** The fluorescence intensity of tumors in the shCHPF group was lower than that of the shCtrl group. **C** The images show the in vivo imaging of tumors in mice in both the shCHPF and shCtrl groups. **D** Images of tumors removed from mice in both the shCHPF and shCtrl groups. **E** The tumors removed from mice in the shCHPF group possessed lower weight than those of the shCtrl group. **F** The effects of CHPF knockdown on the Ki-67 index and cleaved Caspase-3 expression in removed tumor tissues were evaluated by IHC analysis (scale bar = 50 μm). Representative images were selected from at least three independent experiments. The data are expressed as mean ± SD, ^**^P < 0.01.

We further verified the inhibition of gastric cancer by CHPF using the SGC-7901 tumor-bearing mouse xenograft model by subcutaneous injection of SGC-7901 cells transfected with the CHPF vector or the empty vector. A tendency towards a significantly smaller tumor volume was observed in the shCHPF group, which was measured at the indicated time intervals throughout the observation period (*P* < 0.01, Fig. 3A). Additionally, the same trend was also revealed by the in vivo imaging of mouse models, which was consistent with the observation and measurement of the fluorescence intensity of the injected D-luciferin (15 mg/mL) (*P* < 0.01, Fig. 3B, C). The weights of the tumors measured after sacrificing the mice at 28 days post-injection also revealed that smaller tumors were formed by CHPF knockdown cells (Fig. 3D), as illustrated by the data in Fig. 3E. Moreover, as seen in Fig. 3F, the slower growth of tumors in the shCHPF group was further confirmed by the lower Ki-67 index and higher expression of cleaved Caspase-3 detected by IHC analysis. Thus, the inhibition of gastric cancer development by CHPF knockdown was proven in vivo.

### The downstream mechanism underlying the CHPF-induced regulation of gastric cancer was explored by high-throughput sequencing

**Fig. 4:**
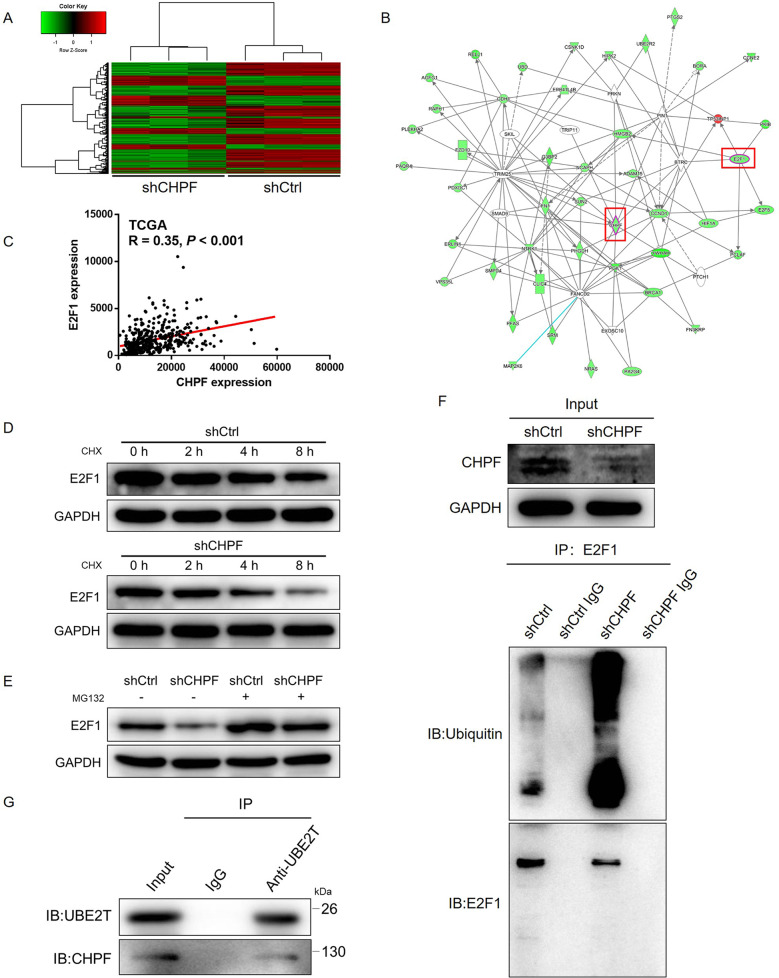
The mechanism of CHPF-induced regulation of gastric cancer. **A** Heatmap of high-throughput sequencing of SGC-7901 cells in the shCtrl and shCHPF groups (3 v 3). **B** IPA was performed to identify the CHPF-related interaction network. **C** The correlation between the expression levels of CHPF and E2F1 in gastric cancer tissues was revealed by analyzing the expression data of TCGA. **D** After the treatment of CHX (0.2 mg/mL), protein stability of E2F1 in shCtrl and shCHPF AGS cells was detected by western blot. **E** The expression of E2F1 in shCtrl and shCHPF AGS cells, with or without MG132 treatment, was detected by western blot. **F** Total protein obtained from different groups of AGS cells was subjected to immunoprecipitation using anti-E2F1 antibody, followed by the detection of ubiquitin. **G** Total protein obtained from AGS cells was subjected to immunoprecipitation using anti-UBE2T antibody, followed by the detection of UBE2T and CHPF by western blot.

To reveal whether CHPF plays a role in the development of gastric cancer in vitro and in vivo, the underlying mechanism was further explored through the detection of the gene expression profile in SGC-7901 cells transfected with shCHPF or shCtrl by high-throughput sequencing (Fig. 4A). In total, 573 differentially expressed genes (DEGs) were identified in the shCHPF group, among which 191 were upregulated and 382 were downregulated (Fig. S2A). Then, the enrichment of the DEGs in canonical signaling pathways and disease and function categories was analyzed by ingenuity pathway analysis (IPA). The results in Fig. S2B show that the most enriched signaling pathway was the p53 signaling pathway, which was consistent with the aforementioned antibody array analysis. Additionally, cancer was identified as the second most enriched disease or function (Fig. S2C). Based on the bioinformatics analysis, various downregulated DEGs were selected as candidates (Fig. S3A) and were subjected to qPCR or WB verification in AGS cells with or without CHPF knockdown, as described in parts B, C, and D of Fig. S3. The results demonstrated significant downregulation of E2F1 at both the mRNA and protein levels in cells with CHPF knockdown. Identical results were obtained from the CHPF-related interaction network analysis, where E2F1 was also revealed to be a potential downstream target of CHPF (Fig. 4B). TCGA expression data also indicated that there was a positive correlation between CHPF and E2F1 (Fig. 4C). The high expression of E2F1 in gastric cancer cells and tissues was also revealed by qPCR (Fig. S3E) and IHC analysis (Fig. S3F), respectively. Notably, based on KM plotter database analysis, high expression of E2F1 was identified as a potential indicator of poor prognosis of gastric cancer (Fig. S3G). All these results suggest that E2F1 and CHPF share a similar role in gastric cancer, with E2F1 acting as a downstream target.

### CHPF regulates E2F1 through UBE2T-mediated ubiquitination

Considering that the stability and activity of E2F1 could be regulated by the ubiquitin–proteasome system (UPS), we evaluated the protein stability of E2F1 protein in AGS cells transfected with shCtrl or shCHPF. As shown in Fig. 4D, knockdown of CHPF distinctly decreased the protein stability of E2F1, as well as downregulated the protein expression of E2F1. Moreover, further detection demonstrated that the regulation of E2F1 protein level by CHPF failed upon MG132 treatment, indicative of the potential involvement of UPS (Fig. 4E). Accordingly, subsequent detection of E2F1 ubiquitylation showed a rising trend in shCHPF cells (Fig. 4F), while E2F1 ubiquitylation showed a decrease in shE2F1 cells (Fig. S3H). In view of the finding, in our previous unpublished work, that E2F1 ubiquitylation could be affected by ubiquitin-conjugating enzyme E2T (UBE2T), we proposed that CHPF may promote the UBE2T-mediated ubiquitylation of E2F1 through interacting UBE2T (Fig. 4G) based on the results of co-IP assay.

### Knockdown of E2F1 impaired the promotion of gastric cancer by CHPF overexpression

**Fig. 5:**
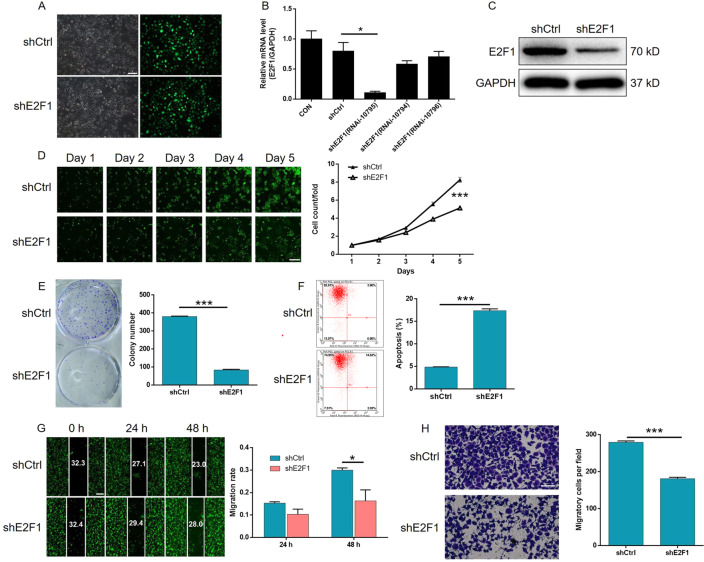
Knockdown of E2F1 inhibited the proliferation and migration and promoted the apoptosis of AGS cells. **A** The efficacy of transfection of AGS cells with shE2F1 and shCtrl was evaluated by fluorescence imaging (scale bar = 100 μm). **B** The efficiency of AGS knockdown by shE2F1 (RNAi-10794, RNAi-10795, and RNAi-10796) was detected by qPCR. **C** The knockdown of E2F1 in AGS cells was detected by western blotting. **D** The effects of E2F1 knockdown on AGS cell proliferation were examined by Celigo cell counting assay (scale bar = 200 μm). **E** The effects of E2F1 knockdown on the colony formation ability of AGS cells were evaluated. **F** The effects of E2F1 knockdown on AGS cell apoptosis were detected by flow cytometry. **G**, **H** The effects of E2F1 knockdown on cell migration ability were estimated by wound-healing assay (**G**, scale bar = 200 μm) and Transwell assay (**H**, scale bar = 200 μm). Representative images were selected from at least three independent experiments. The data are expressed as mean ± SD, ^*^P < 0.05, ^***^P < 0.001.

To clarify whether E2F1 acts downstream of CHPF in the regulation of gastric cancer, constructs with only CHPF overexpression, only E2F1 knockdown, and simultaneous CHPF overexpression and E2F1 knockdown were transfected into AGS and SGC-7901 cells. First, the efficiencies of transfection and CHPF overexpression were detected by fluorescence imaging (Figs. S4A and S5A), qPCR (Figs. S4B and S5B), and WB (Figs. S4C and S5C), as mentioned previously. As expected, significant effects caused by the overexpression of CHPF were detected, including the promotion of cell proliferation (detected by the Celigo cell counting assay) (*P* < 0.001, Figs. S4D and S5D) and colony formation (*P* < 0.001, Figs. S4E and S5E) and the inhibition of cell apoptosis (*P* < 0.001, Figs. S4F and S5F), which was precisely opposite to the effects of CHPF knockdown. Moreover, the results of the wound-healing assay were consistent with those of the Transwell assay, in which the migration ability of gastric cancer cells appeared to be promoted by CHPF overexpression (*P* < 0.001, Figs. S4G, H and S5G, H). On the other hand, based on the transfection (Figs. 5A and S6A) and qPCR results, RNAi-10795 was used for E2F1 knockdown in all subsequent experiments (Fig. 5B). After the knockdown of E2F1 was confirmed by qPCR and WB (Figs. 5B, C and S6B, C), subsequent assays showed that it exhibited similar effects as the knockdown of CHPF, inhibiting cell growth and colony formation, suppressing cell migration, and promoting cell apoptosis (*P* < 0.05, Figs. 5D–H and S6D–H). More importantly, knocking down the expression of E2F1 in CHPF-overexpressing AGS and SGC-7901 cells significantly alleviated or even reversed the promotion of cell proliferation, colony formation, and cell migration and the inhibition of cell apoptosis by CHPF overexpression (*P* < 0.05, Figs. S7–S9 and 6A–J).

**Fig. 6:**
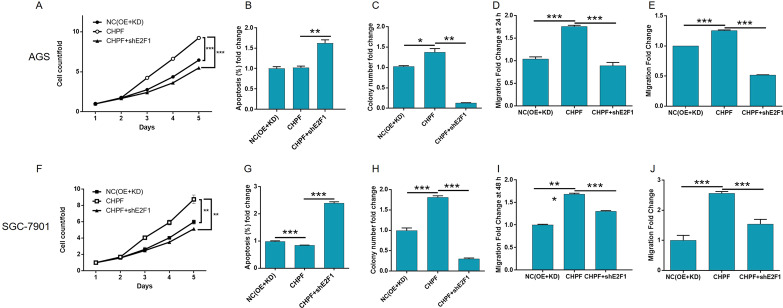
E2F1 knockdown alleviates the effects of CHPF overexpression on gastric cancer cells. The influence of E2F1 knockdown on the CHPF overexpression induced changes in proliferation, apoptosis, colony formation, and migration of AGS (**A**–**E**) and SGC-7901 (**F**–**J**) cells was detected by Celigo cell counting assay (**A**, **F**), flow cytometry (**B**, **G**), colony formation assay (**C**, **H**), wound-healing assay (**D**, **I**), and Transwell assay (**E**, **J**), respectively. All data were collected from at least three independent experiments and were normalized to the corresponding negative control. The data are expressed as mean ± SD, ^**^P < 0.01, ^***^P < 0.001.

## Discussion

Gastric cancer is one of the most common digestive system tumors and is one of the most lethal tumors worldwide. At present, surgical resection is the main treatment for patients with early gastric cancer, while chemotherapy is the preferred treatment for patients with advanced gastric cancer [15, 16]. However, given that the symptoms are nonspecific, most patients with gastric cancer are diagnosed at advanced stages [17]. Therefore, enhancing the effects of chemotherapy by seeking new specific therapeutic targets is important for improving the prognosis of gastric cancer. Recently, a number of studies demonstrated that molecular biology and gene technology advancements such as high-throughput sequencing have offered new opportunities to research the molecular mechanisms underlying gastric cancer, and various novel targets have been discovered [18–21]. For example, Liu et al. noted that a potential tumor promoter, NETO2, was upregulated in gastric cancer tissues and could promote the invasion and metastasis of gastric cancer through TNFRSF12A-mediated activation of the PI3K/AKT/NF-κB/Snail axis [22]. It has recently been found that KLF9 was able to inhibit the migration and invasion of gastric cancer cells and suppress tumor metastasis in vivo, which may be associated with the transcriptional regulation of MMP28 [23].

CHPF is a type II transmembrane protein that plays a critical role in the biosynthesis of CS [10]. CHPF, which is abnormally expressed in human cancer, can act as a potential tumor promoter. In a recent study by Hou et al., the role of CHPF in lung adenocarcinoma (LUAD) was investigated, and the results indicated that CHPF could accelerate cell growth and inhibit apoptosis in LUAD cells. Mechanistic exploration demonstrated the potential involvement of the MAPK signaling pathway in the CHPF-mediated promotion of LUAD [24]. Fan et al. reported the effects of CHPF knockdown on glioma cell behavior, including inhibiting proliferation, promoting apoptosis and affecting the cell cycle, suggesting the potential role of CHPF in glioma [14]. In addition, CHPF has been observed to be abnormally expressed in colorectal cancer and head and neck squamous cell cancer [12, 13]. Despite all this research progress, cancer-related studies of CHPF are still rare, and the relationship between CHPF and gastric cancer has not yet been established.

This study is the first comprehensive investigation of the upregulated expression of CHPF in gastric cancer tissues in comparison with normal tissues. Through analysis of the prognosis of 876 patients in the KM plotter database, the association between high CHPF expression and T infiltrate, advanced tumor stage and poor survival rate was determined. It should be noted that knockdown of CHPF in gastric cancer cells inhibited cell proliferation and colony formation, promoted apoptosis through the regulation of apoptosis-related biomarkers, and suppressed the migration ability of the cells. Simultaneously, the promotive effects of CHPF overexpression on the development and progression of gastric cancer were also proven. In addition, a mouse xenograft model constructed with SGC-7901 cells with or without CHPF knockdown was used to conduct in vivo experiments where the impaired tumor growth and downregulated expression of Ki-67 by CHPF knockdown were illustrated. To the best of our knowledge, this is the first report that demonstrates the promotive effects of CHPF on gastric cancer in vitro and in vivo. Moreover, the underlying mechanism of the CHPF-induced regulation of gastric cancer was explored through RNA sequencing followed by bioinformatics analysis. From the results we obtained on the enrichment of DEGs and the analysis of the CHPF-related interaction network, E2F1 was identified as a promising downstream target of CHPF.

It is well known that E2F1 is the earliest and most widely studied member in the E2F family of transcription factors [25]. The transcription factor E2F1 is involved in the regulation of cell proliferation, differentiation and apoptosis and plays an important role in the regulation of G1–S phase transition in the cell cycle [25–27]. Much work thus far has shown that the elevated expression of E2F1 can lead to the abnormal growth of cells and that it can participate in the occurrence and development of malignant tumors [28, 29]. At the same time, several excellent studies have described the abnormally upregulated expression level of E2F1 in lung cancer and breast cancer [30]. A study on the association of p53 and p73 with the regulation of cell apoptosis by E2F1 was reported by Polager et al. [31]. Correspondingly, the E2F1-p73-apoptosis axis was reported to be involved in p27T187A knock-in-mediated regulation of advanced prostate cancer [32]. A recent study also demonstrated that the downregulation of E2F1 in melanoma cells could induce cell senescence and cell death and further increase the sensitivity of melanoma cells to BRAF inhibitors [33]. In addition, the role of E2F1 in various types of cancer, such as renal carcinoma [34], colorectal cancer [35], bladder cancer [36], and ovarian cancer [37], has been investigated. More importantly, the role of E2F1 in gastric cancer has been extensively studied. For example, it was reported that the posttranscriptional regulation of the miR-106b-25 cluster by E2F1 promoted TGF-β resistance in gastric cancer [38]. Considerable research efforts by Xu et al. proved that E2F1 exerted its tumor-promotive effects on gastric cancer based on the regulation of TINCR transcriptional activity and the TINCR/STAU1/CDKN2B signaling pathway [39]. Through high-throughput sequencing of SGC-7901 cells with or without CHPF knockdown, the present study identified E2F1 as a potential downstream effector of CHPF and revealed the possible mechanism by which CHPF regulates gastric cancer. Further construction of a cell model with stable knockdown of E2F1 followed by cell function assays revealed the similar effects of E2F1 knockdown and CHPF knockdown on the development and progression of gastric cancer, including the inhibition of cell proliferation and colony formation, the suppression of migration ability, and the promotion of cell apoptosis. Furthermore, it was also clarified that the effects of CHPF overexpression on the proliferation, colony formation, apoptosis, and migration of gastric cancer cells was significantly alleviated by the simultaneous knockdown of E2F1. On the basis of these results, one could conclude that CHPF may promote the development of gastric cancer through the regulation of E2F1.

Ubiquitination refers to the process of specific modification of target proteins by ubiquitin molecules under the action of a series of special enzymes. Ubiquitination plays an important role in protein localization, metabolism, function, regulation, and degradation. At the same time, it is also involved in the regulation of almost all life activities such as cell cycle, proliferation, apoptosis, differentiation, metastasis, gene expression, transcriptional regulation, signal transmission, damage repair, and inflammatory immunity. Ubiquitination is closely related to the pathogenesis of diseases such as malignant tumors. Previously, it has been reported that E2F1 expression could be regulated by UPS in glioma and liver cancer [40, 41]. Our study also demonstrated that knockdown of CHPF downregulated E2F1 expression through promoting ubiquitination. Furthermore, we identified UBE2T as a key member in the UPS-mediated degradation of E2F1 and verified the interaction between UBE2T and CHPF. Therefore, it was reasonable to deduce that CHPF may interact with UBE2T, thus affecting the UBE2T-mediated E2F1 ubiquitination and regulating E2F1 expression.

In summary, the upregulated expression of CHPF in gastric cancer tissues and the positive correlation between high expression of CHPF and poor prognosis were revealed in this study. Further investigations showed that CHPF may promote the proliferation, colony formation, and migration and inhibit the apoptosis of gastric cancer cells through the regulation of E2F1. Therefore, CHPF may act as an oncogene-like protein and serve as a prognostic indicator and therapeutic target in gastric cancer treatment.

## Supplementary information

Table S1.

Table S2.

Table S3.

Supplementary figure legends.

Figure S1.

Figure S2.

Figure S3.

Figure S4.

Figure S5.

Figure S6.

Figure S7.

Figure S8.

Figure S9.

## Data Availability

All data generated or analyzed during this study are included in this published article and its supplementary information files.

## References

[1] Siegel RL, Miller KD, Fuchs HE, Jemal A (2021). Cancer Statistics, 2021. CA Cancer J Clin.

[2] Bray F, Ferlay J, Soerjomataram I, Siegel RL, Torre LA, Jemal A (2018). Global cancer statistics 2018: GLOBOCAN estimates of incidence and mortality worldwide for 36 cancers in 185 countries. CA Cancer J Clin.

[3] Orditura M, Galizia G, Sforza V, Gambardella V, Fabozzi A, Laterza MM (2014). Treatment of gastric cancer. World J Gastroenterol.

[4] Coevorden FV, Vanhoutvin S, Hulshof MC, Loosveld OJ, Tije AJT, Erdkamp FL (2018). Chemotherapy versus chemoradiotherapy after surgery and preoperative chemotherapy for resectable gastric cancer (CRITICS): an international, open-label, randomised phase 3 trial. Lancet Oncol.

[5] Sitarz R, Skierucha M, Mielko J, Offerhaus GJA, Maciejewski R, Polkowski WP (2018). Gastric cancer: epidemiology, prevention, classification, and treatment. Cancer Manag Res.

[6] Guggenheim DE, Shah MA (2013). Gastric cancer epidemiology and risk factors. J Surg Oncol.

[7] Fujita T (2012). Targeted therapy for gastric cancer. Lancet Oncol.

[8] Tadahisa M, Hiroshi K (2013). Biosynthesis and function of chondroitin sulfate. Biochim Biophys Acta.

[9] Hiroyasu O, Masafumi S, Nobuo S, Sonoko H, Naoko N, Yukihiko K (2010). Chondroitin sulfate synthase-2/chondroitin polymerizing factor has two variants with distinct function. J Biol Chem.

[10] Hiroshi K, Tomomi I, Toru U, Kazuyuki S (2003). Molecular cloning of a chondroitin polymerizing factor that cooperates with chondroitin synthase for chondroitin polymerization. J Biol Chem.

[11] Ogawa H, Hatano S, Sugiura N, Nagai N, Sato T, Shimizu K (2012). Chondroitin sulfate synthase-2 is necessary for chain extension of chondroitin sulfate but not critical for skeletal development. PLoS ONE.

[12] Kalathas D, Theocharis DA, Bounias D, Kyriakopoulou D, Papageorgakopoulou N, Stavropoulos MS (2011). Chondroitin synthases I, II, III and chondroitin sulfate glucuronyltransferase expression in colorectal cancer. Mol Med Rep..

[13] Muy-Teck T, Emilios G, Deeviyaben P, Rameez T, Ayesha N, Bahta AW (2012). FOXM1 induces a global methylation signature that mimics the cancer epigenome in head and neck squamous cell carcinoma. PLoS ONE.

[14] Fan Y, Xiao B, Lv S, Ye M, Zhu X, Wu M (2017). Lentivirus‑mediated knockdown of chondroitin polymerizing factor inhibits glioma cell growth in vitro. Oncol Rep..

[15] Tan Z (2019). Recent advances in the surgical treatment of advanced gastric cancer: a review. Med Sci Monit.

[16] Bonelli P, Borrelli A, Tuccillo FM, Silvestro L, Palaia R, Buonaguro FM (2019). Precision medicine in gastric cancer. World J Gastrointest Oncol.

[17] Fukuda N, Sugiyama Y, Wada J (2011). Prognostic factors of T4 gastric cancer patients undergoing potentially curative resection. World J Gastroenterol.

[18] Anders S, Pyl PT, Huber W (2015). HTSeq-a Python framework to work with high-throughput sequencing data. Bioinformatics.

[19] Tian P, Liang C (2018). Transcriptome profiling of cancer tissues in Chinese patients with gastric cancer by high-throughput sequencing. Oncol Lett.

[20] Li P, Chen H, Chen S, Mo X, Li T, Xiao B (2017). Circular RNA 0000096 affects cell growth and migration in gastric cancer. Br J Cancer.

[21] Wang X, Che X, Liu C, Fan Y, Bai M, Hou K (2018). Cancer-associated fibroblasts-stimulated interleukin-11 promotes metastasis of gastric cancer cells mediated by upregulation of MUC1. Exp Cell Res.

[22] Liu J, Jiang L, He T, Liu J, Fan J, Xu X (2019). NETO2 promotes invasion and metastasis of gastric cancer cells via activation of PI3K/Akt/NF-κB/Snail axis and predicts outcome of the patients. Cell Death Dis.

[23] Li Y, Sun Q, Jiang M, Li S, Zhang J, Xu Z (2019). KLF9 suppresses gastric cancer cell invasion and metastasis through transcriptional inhibition of MMP28. FASEB J.

[24] Hou X, Zhang T, Da Z, Wu X (2019). CHPF promotes lung adenocarcinoma proliferation and anti-apoptosis via the MAPK pathway. Pathol Res Pract.

[25] Bell LA, Ryan KM (2004). Life and death decisions by E2F-1. Cell Death Differ.

[26] Jiang H, Martin V, Alonso M, Gomezmanzano C, Fueyo J (2010). RB-E2F1: molecular rheostat for autophagy and apoptosis. Autophagy.

[27] Wang C, Rauscher FJ, Cress WD, Chen J (2007). Regulation of E2F1 function by the nuclear corepressor KAP1. J Biol Chem.

[28] Louie MC, Zou JX, Rabinovich A, Chen HW (2004). ACTR/AIB1 functions as an E2F1 coactivator to promote breast cancer cell proliferation and antiestrogen resistance. Mol Cell Biol.

[29] Berteaux N, Lottin S, Montã D, Pinte S, Quatannens B, Coll J (2005). H19 mRNA-like noncoding RNA promotes breast cancer cell proliferation through positive control by E2F1. J Biol Chem.

[30] Panayotis Z, Athanassios K, Konstantinos E, Panagiotis K, Leandros-V V, Nousin R (2004). Distinct expression patterns of the transcription factor E2F-1 in relation to tumour growth parameters in common human carcinomas. J Pathol.

[31] Polager S, Ginsberg D (2008). E2F – at the crossroads of life and death. Trends Cell Biol.

[32] Zhao H, Lu Z, Bauzon F, Fu H, Cui J, Locker J (2016). p27T187A knockin identifies Skp2/Cks1 pocket inhibitors for advanced prostate cancer. Oncogene.

[33] Rouaud F, Hamoudatekaya N, Cerezo M, Abbe P, Zangari J, Hofman V (2018). E2F1 inhibition mediates cell death of metastatic melanoma. Cell Death Dis.

[34] Ding M, Lu X, Wang C, Zhao Q, Ge J, Xia Q (2018). The E2F1-miR-520/372/373-SPOP axis modulates progression of renal carcinoma. Cancer Res.

[35] Dosil MA, Navaridas R, Mirantes C, Tarragona J, Eritja N, Felip I (2019). Tumor suppressive function of E2F-1 on PTEN-induced serrated colorectal carcinogenesis. J Pathol.

[36] Su F, He W, Chen C, Liu M, Liu H, Xue F (2018). The long non-coding RNA FOXD2-AS1 promotes bladder cancer progression and recurrence through a positive feedback loop with Akt and E2F1. Cell Death Dis.

[37] Zhang X, Liu G, Qiu J, Ning Z, Ding J, Hua K (2017). E2F1-regulated long non-coding RNA RAD51-AS1 promotes cell cycle progression, inhibits apoptosis and predicts poor prognosis in epithelial ovarian cancer. Sci Rep.

[38] Petrocca F, Visone R, Onelli MR, Shah MH, Nicoloso MS, de Martino I (2008). E2F1-regulated microRNAs impair TGFβ-dependent cell-cycle arrest and apoptosis in gastric cancer. Cancer Cell.

[39] Xu T, Wang Y, Xiong W, Ma P, Wang W, Chen W (2017). E2F1 induces TINCR transcriptional activity and accelerates gastric cancer progression via activation of TINCR/STAU1/CDKN2B signaling axis. Cell Death Dis.

[40] Wang B, Ma A, Zhang L, Jin W, Qian Y, Xu G (2015). POH1 deubiquitylates and stabilizes E2F1 to promote tumour formation. Nat Commun.

[41] Zhi T, Jiang K, Xu X, Yu T, Zhou F, Wang Y (2019). ECT2/PSMD14/PTTG1 axis promotes the proliferation of glioma through stabilizing E2F1. Neuro-Oncol.

