# Supplementary material for: CHPF promotes gastric cancer tumorigenesis through the activation of E2F1
Source: Cell Death Dis. 2021 Sep 25;12(10):876. doi: 10.1038/s41419-021-04148-y (PMC8464597; doi:10.1038/s41419-021-04148-y)
Supplement: Supplementary file 1 — Table S1. [file 41419_2021_4148_MOESM1_ESM.docx]

Table S1 Primers used for qPCR

| Gene | Forward primer (5’ to 3’) | Reverse primer (5’ to 3’) |
| --- | --- | --- |
| CHPF | GGAACGCACGTACCAGGAG | CGGGATGGTGCTGGAATACC |
| CCNE2 | TTGGCTATGCTGGAGGAAGTAA | TTCAGTGCTCTTCGGTGGTG |
| CDKN1A | TCTTGTACCCTTGTGCCTCG | GAAATCTGTCATGCTGGTCTGC |
| E2F8 | TGACGAAGTGGCAGAGGAAC | CATCATAATCTGCTCGGCGTA |
| MET | AATCTTGGGACATCAGAGGGTC | GGATGGGAGTCCAGGAGAAAA |
| PAK1 | TGTCACAGGGGAGTTTACGG | CTGGCTGTTGGATGTCTTCTTC |
| NRAS | AAACCTCAGCCAAGACCAGA | AACCCTGAGTCCCATCATCAC |
| ETS2 | CGGGGCTTACGCTACTATTACG | CAAGTTCTGGAGGTCGCACA |
| PLCG1 | GCCTATGCAGATGAACCAGG | CATTCTTTGGCAGATGTCGG |
| PIK3CB | CTGCGACAGATGAGTGATGAAG | CCCTATCCTCCGATTACCAAG |
| PTGS2 | CAAATCCTTGCTGTTCCCACC | TTTCTCCATAGAATCCTGTCCG |
| HDAC9 | GCTGGTGGAGTTCCCTTACAT | AAAGGTGCAGACTGGGTTCG |
| TIGAR | GGTGAAAATGCGTGGAATAGAC | CTTGGAGATCCTTGGGAAAACT |
| CSNK1D | GCTGCTTGCTGACCAAATGA | GAGGAAGTTGTCTGGCTTCACA |
| HIF1A | AGCCGAGGAAGAACTATGAACA | TCACAAATCAGCACCAAGCAG |
| FAS | GGACATGGCTTAGAAGTGGAAA | ACTTGGTGTTGCTGGTGAGTG |
| TP53I3 | TTTGCTGAGGTCTAGGGACAAT | TGGATTTCGGTCACTGGGTAG |
| TRIM29 | CAAGGAGACCACCCAGAAGAA | GGGCAGGTCATTGTCAGAGTT |
| HIPK2 | TCCAACTGGGACATGACTGG | CGATGGTCTGCTCGTAAGGTA |
| E2F1 | CACTTTCGGCCCTTTTGCTC | GTGCTCTCACCGTCCTACAC |
| CCND1 | AGCTGTGCATCTACACCGAC | GAAATCGTGCGGGGTCATTG |
| GAPDH | TGACTTCAACAGCGACACCCA | CACCCTGTTGCTGTAGCCAAA |
