# Supplementary material for: CHPF promotes gastric cancer tumorigenesis through the activation of E2F1
Source: Cell Death Dis. 2021 Sep 25;12(10):876. doi: 10.1038/s41419-021-04148-y (PMC8464597; doi:10.1038/s41419-021-04148-y)
Supplement: Supplementary file 2 — Table S2. [file 41419_2021_4148_MOESM2_ESM.docx]

Table S2 Antibodies used in western blotting and IHC

| Primary antibodies | Dilution in WB | Source species | Company | Catalog No. |
| --- | --- | --- | --- | --- |
| CHPF | 1:1000 in WB  1:200 in IHC | Rabbit | Abcam | ab224495 |
| COX2 | 1:500 | Rabbit | BOSTER | BA0738 |
| Cyclin D1 | 1:2000 | Rabbit | CST | 2978 |
| E2F1 | 1:1000 in WB  1:100 in IHC | Rabbit | Abcam | ab179445 |
| P21 | 1:200 | Rabbit | BOSTER | BM3990 |
| P27 | 1:200 | Rabbit | BOSTER | BM4229 |
| UBE2T | 1:2000 | Rabbit | Proteintech | 10105-2-AP |
| Ubiquitin | 1:750 | Rabbit | Proteintech | 10201-2-AP |
| GAPDH | 1:3000 | Rabbit | Bioworld | AP0063 |
|  |  |  |  |  |
| Secondary antibody | Dilution |  | Company | Catalog No. |
| HRP Goat Anti-Rabbit IgG (WB) | 1:3000 |  | Beyotime | A0208 |
| HRP Goat Anti-Rabbit IgG (IHC) | 1:400 |  | Abcam | ab6721 |
