# Supplementary material for: CHPF promotes gastric cancer tumorigenesis through the activation of E2F1
Source: Cell Death Dis. 2021 Sep 25;12(10):876. doi: 10.1038/s41419-021-04148-y (PMC8464597; doi:10.1038/s41419-021-04148-y)
Supplement: Supplementary file 3 — Table S3. [file 41419_2021_4148_MOESM3_ESM.docx]

Table 3 Relationship between CHPF expression and tumor characteristics in patients with gastric cancer analyzed by Pearson correlation analysis

| Tumor characteristics | index |  |
| --- | --- | --- |
| T [Infiltrate](D:/360%E5%AE%89%E5%85%A8%E6%B5%8F%E8%A7%88%E5%99%A8%E4%B8%8B%E8%BD%BD/Dict/8.4.0.0/resultui/html/index.html#/javascript:;) | Pearson correlation | 0.172 |
|  | Significance (two tailed) | 0.043 |
|  | n | 139 |
| Stage | Pearson correlation | 0.214 |
|  | Significance (two tailed) | 0.012 |
|  | n | 139 |
