# Supplementary material for: CHPF promotes gastric cancer tumorigenesis through the activation of E2F1
Source: Cell Death Dis. 2021 Sep 25;12(10):876. doi: 10.1038/s41419-021-04148-y (PMC8464597; doi:10.1038/s41419-021-04148-y)
Supplement: Supplementary file 4 — Supplementary figure legends. [file 41419_2021_4148_MOESM4_ESM.docx]

**Fig. S1.** (A) mRNA expression of CHPF in GES1 cells and gastric cancer cell lines including AGS, BGC-823, SGC-7901 and MGC-803 by qPCR. (B) The transfection efficiencies of shCtrl and shCHPF in AGS and SGC-7901 cells were evaluated by fluorescence imaging.

**Fig. S2.** (A) Scatter plot and volcano plot showed the threshold in identification of differentially expressed genes (DEGs) after high throughput sequencing. IPA analysis was performed to identify the canonical pathways (B) and disease & functions (C) enriched by the DEGs.

**Fig. S3.** (A) The partial heatmap of the selected DEGs in high throughput sequencing of SGC-7901 cells in shCtrl and shCHPF groups (3 v 3). (B, C) The mRNA expression of selected DEGs in AGS cells with or without CHPF knockdown was detected by qPCR. (D) The protein levels of selected DEGs in AGS cells with or without CHPF knockdown was detected by western blotting. (E) mRNA expression of E2F1 in GES1 cells and gastric cancer cell lines including AGS, BGC-823, SGC-7901 and MGC-803 by qPCR. (F) The expression of E2F1 in gastric cancer tissues and normal tissues was detected by IHC analysis. (G) The relationship between E2F1 expression and gastric cancer prognosis was displayed by data mining of the KM plotter database. (H) Total protein obtained from different groups of AGS cells was subjected to immunoprecipitation using anti-E2F1 antibody, followed by the detection of ubiquitin. Representative images were selected from at least three independent experiments. The data were expressed as mean ± SD, **P*<0.05, ***P*<0.01, ****P*<0.001.

**Fig. S4.** **Over-expression of CHPF promoted proliferation, migration and inhibited apoptosis of AGS cells.** (A) The efficacy of transfection in AGS cells by lentivirus for CHPF over-expression (CHPF) or negative control (Control) was evaluated by fluorescence imaging. (B) The efficiency of over-expression in AGS was detected by qPCR. (C) The over-expression of CHPF in AGS cells was detected by western blotting. (D) The effects of CHPF over-expression on cell proliferation of AGS cells were examined by Celigo cell counting assay. (E) The effects of CHPF over-expression on colony formation ability of AGS cells were evaluated. (F) The effects of CHPF over-expression on cell apoptosis of AGS cells were detected by flow cytometry. (G, H) The effects of CHPF over-expression on cell migration ability were estimated by wound-healing (G) and Transwell assays (H). The representative images were selected from at least three independent experiments. The data were expressed as mean ± SD, ***P*<0.01, ****P*<0.001.

**Fig. S5. Over-expression of CHPF promoted proliferation, migration and inhibited apoptosis of SGC-7901 cells.** (A) The efficacy of transfection in SGC-7901 cells by lentivirus for CHPF over-expression (CHPF) or negative control (Control) was evaluated by fluorescence imaging. (B) The efficiency of over-expression in SGC-7901 was detected by qPCR. (C) The over-expression of CHPF in SGC-7901 cells was detected by western blotting. (D) The effects of CHPF over-expression on cell proliferation of SGC-7901 cells were examined by Celigo cell counting assay. (E) The effects of CHPF over-expression on colony formation ability of SGC-7901 cells were evaluated. (F) The effects of CHPF over-expression on cell apoptosis of SGC-7901 cells were detected by flow cytometry. (G, H) The effects of CHPF over-expression on cell migration ability were estimated by wound-healing (G) and Transwell assays (H). The representative images were selected from at least three independent experiments. The data were expressed as mean ± SD, ***P*<0.01, ****P*<0.001.

**Fig. S6. Knockdown of E2F1 inhibited proliferation, migration and promoted apoptosis of SGC-7901 cells.** (A) The efficacy of transfection in SGC-7901 cells by shE2F1 and shCtrl was evaluated by fluorescence imaging. (B) The efficiency of knockdown in SGC-7901 by shE2F1 was detected by qPCR. (C) The knockdown of E2F1 in SGC-7901 cells was detected by western blotting. (D) The effects of E2F1 knockdown on cell proliferation of SGC-7901 cells were examined by Celigo cell counting assay. (E) The effects of E2F1 knockdown on colony formation ability of SGC-7901 cells were evaluated. (F) The effects of E2F1 knockdown on cell apoptosis of SGC-7901 cells were detected by flow cytometry. (G, H) The effects of E2F1 knockdown on cell migration ability were estimated by wound-healing (G) and Transwell assays (H). The representative images were selected from at least three independent experiments. The data were expressed as mean ± SD, **P*<0.05, ****P*<0.001.

**Fig. S7.** The expression levels of CHPF and E2F1 in AGS and SGC-7901 cells with simultaneously CHPF over-expression and E2F1 knockdown were detected by qPCR (A) and western blotting (B), respectively.

**Fig. S8.** The influence of E2F1 knockdown on the CHPF overexpression-induced changes in proliferation, apoptosis, colony formation and migration of AGS cells was detected by Celigo cell counting assay (A), flow cytometry (B), colony formation assay (C), wound-healing assay (D) and Transwell assay (E), respectively.

**Fig. S9.** The influence of E2F1 knockdown on the CHPF overexpression-induced changes in proliferation, apoptosis, colony formation and migration of SGC-7901 cells was detected by Celigo cell counting assay (A), flow cytometry (B), colony formation assay (C), wound-healing assay (D) and Transwell assay (E), respectively.
