# Supplementary figures and images for: CHPF promotes gastric cancer tumorigenesis through the activation of E2F1
Source: Cell Death Dis. 2021 Sep 25;12(10):876. doi: 10.1038/s41419-021-04148-y (PMC8464597; doi:10.1038/s41419-021-04148-y)

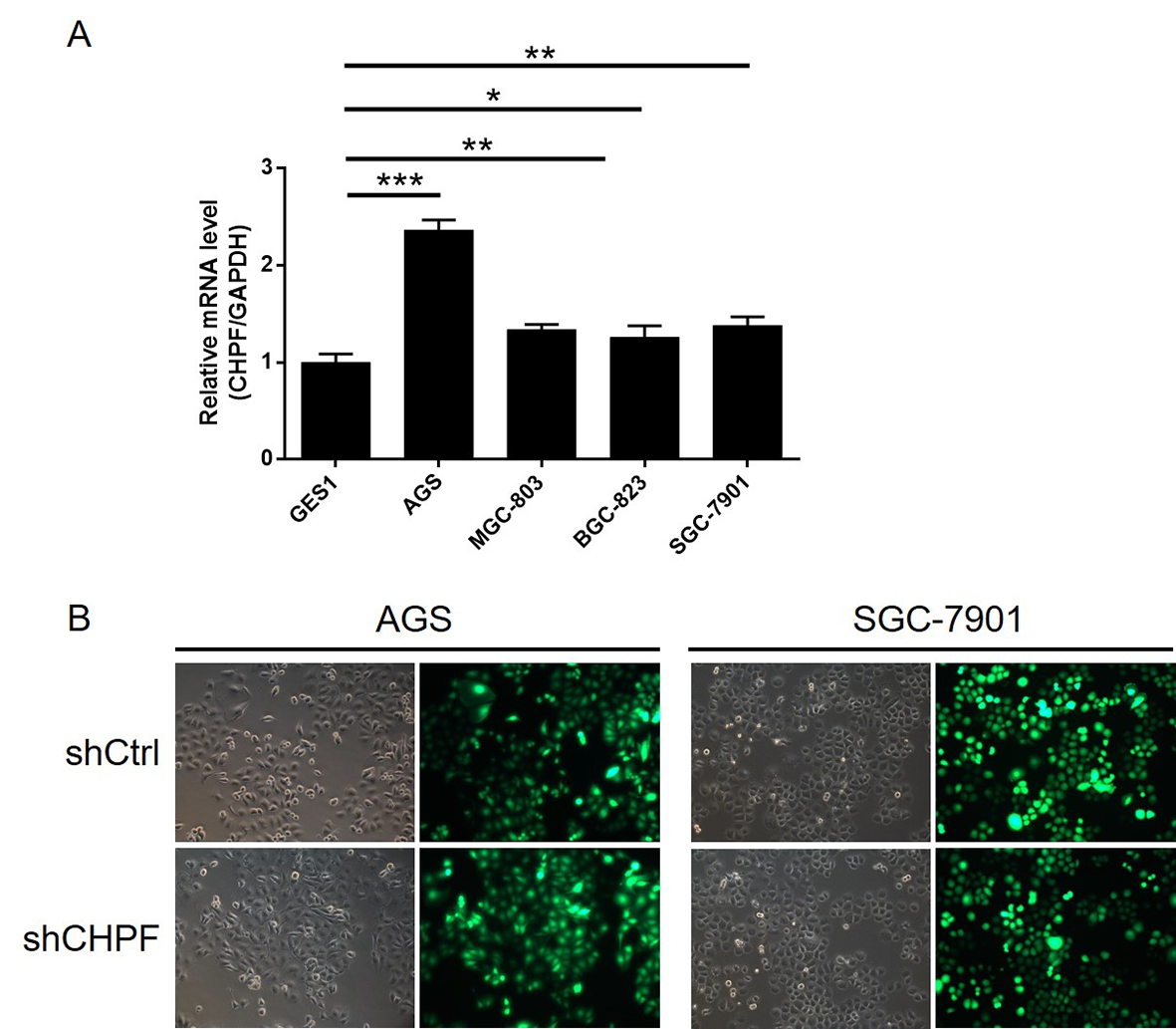

Supplement: Supplementary file 5 — Figure S1. [file 41419_2021_4148_MOESM5_ESM.tif]

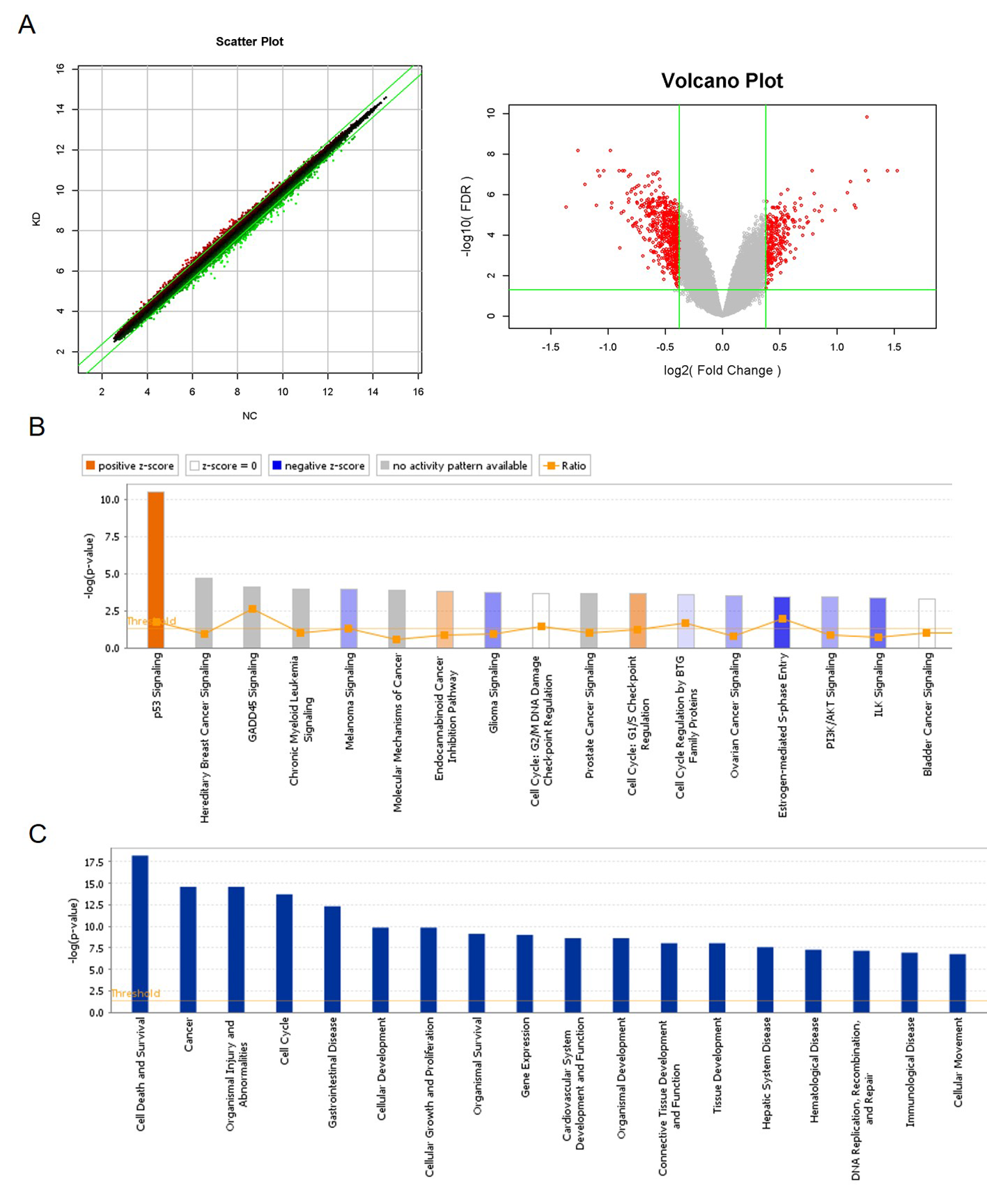

Supplement: Supplementary file 6 — Figure S2. [file 41419_2021_4148_MOESM6_ESM.tif]

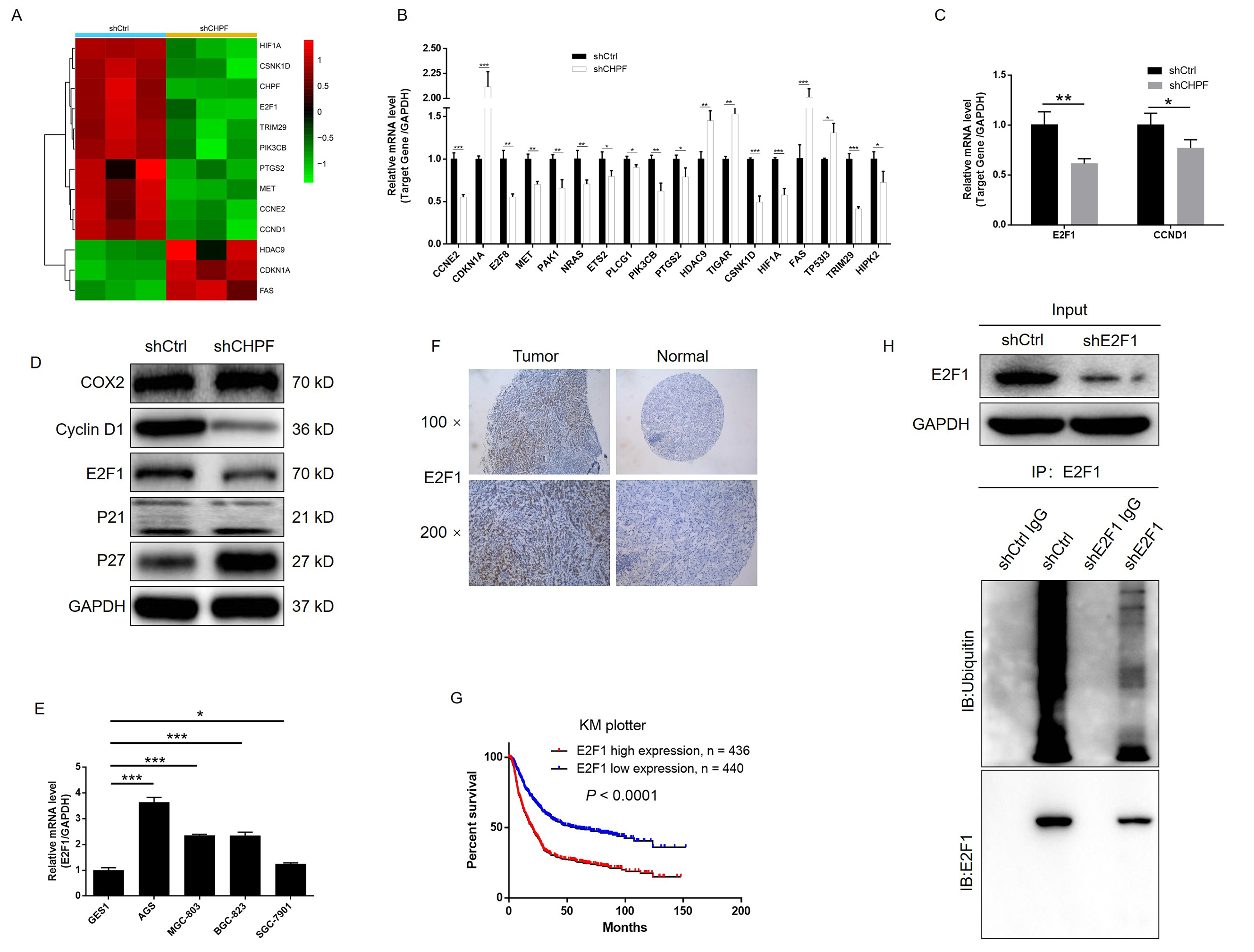

Supplement: Supplementary file 7 — Figure S3. [file 41419_2021_4148_MOESM7_ESM.tif]

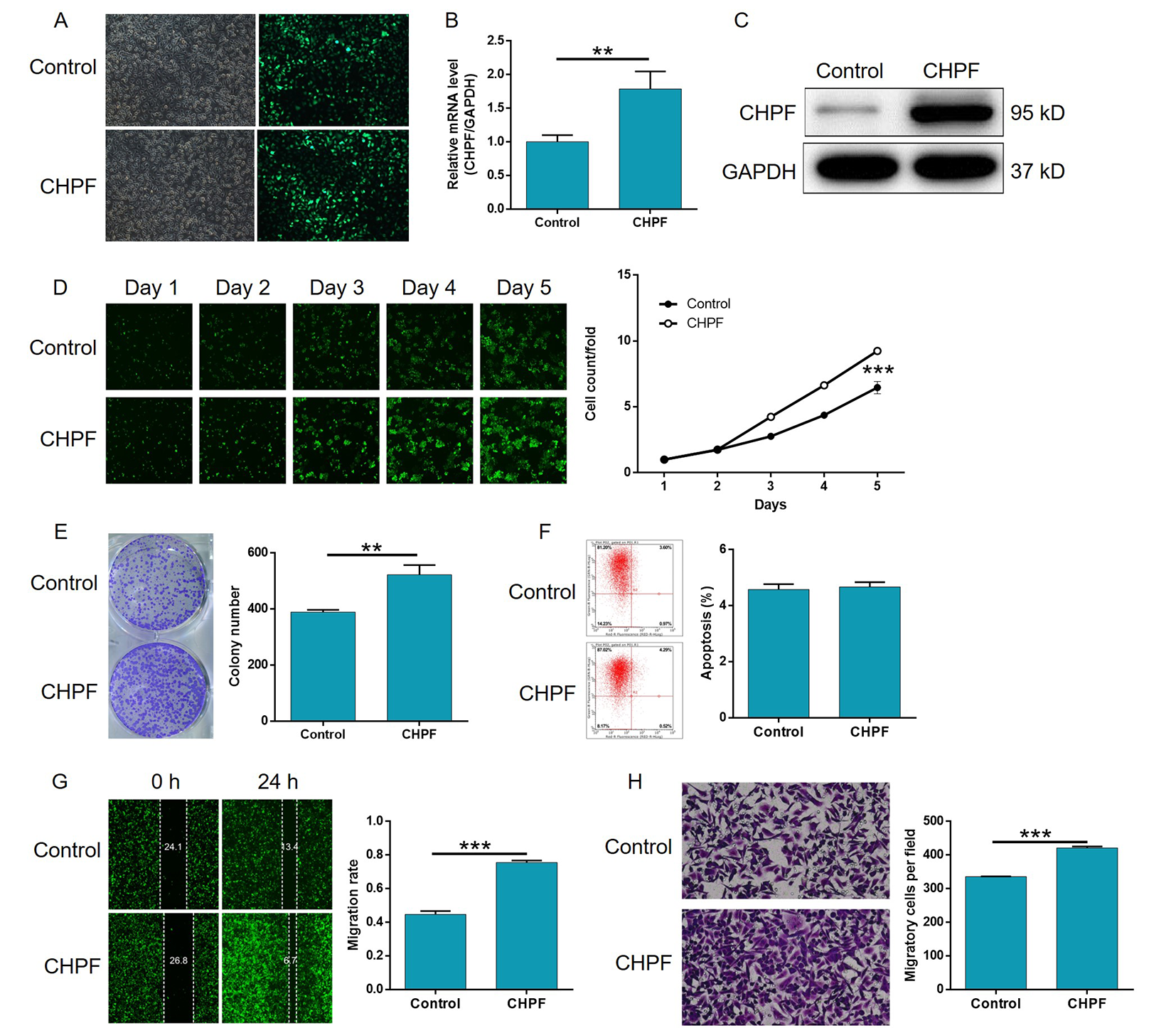

Supplement: Supplementary file 8 — Figure S4. [file 41419_2021_4148_MOESM8_ESM.tif]

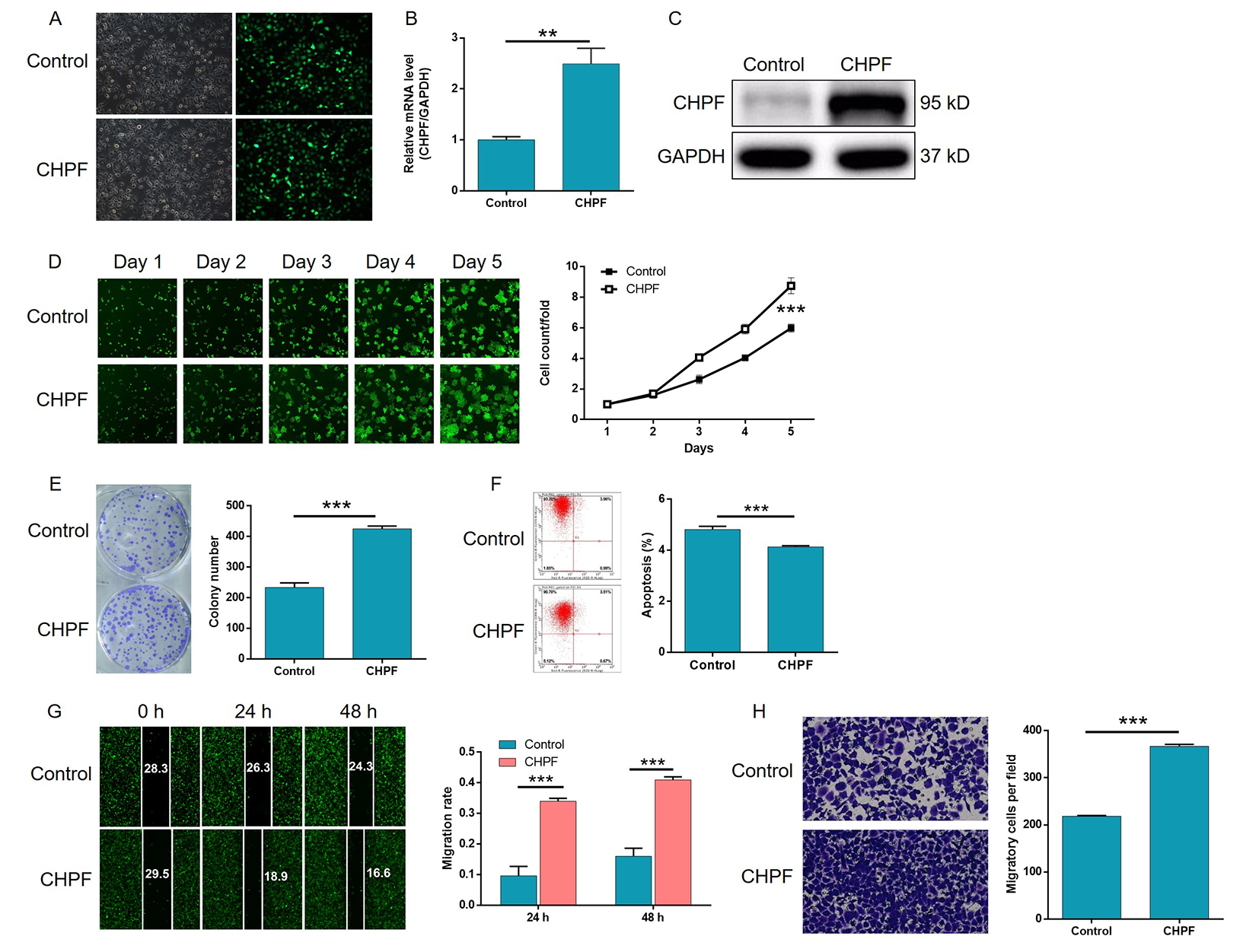

Supplement: Supplementary file 9 — Figure S5. [file 41419_2021_4148_MOESM9_ESM.tif]

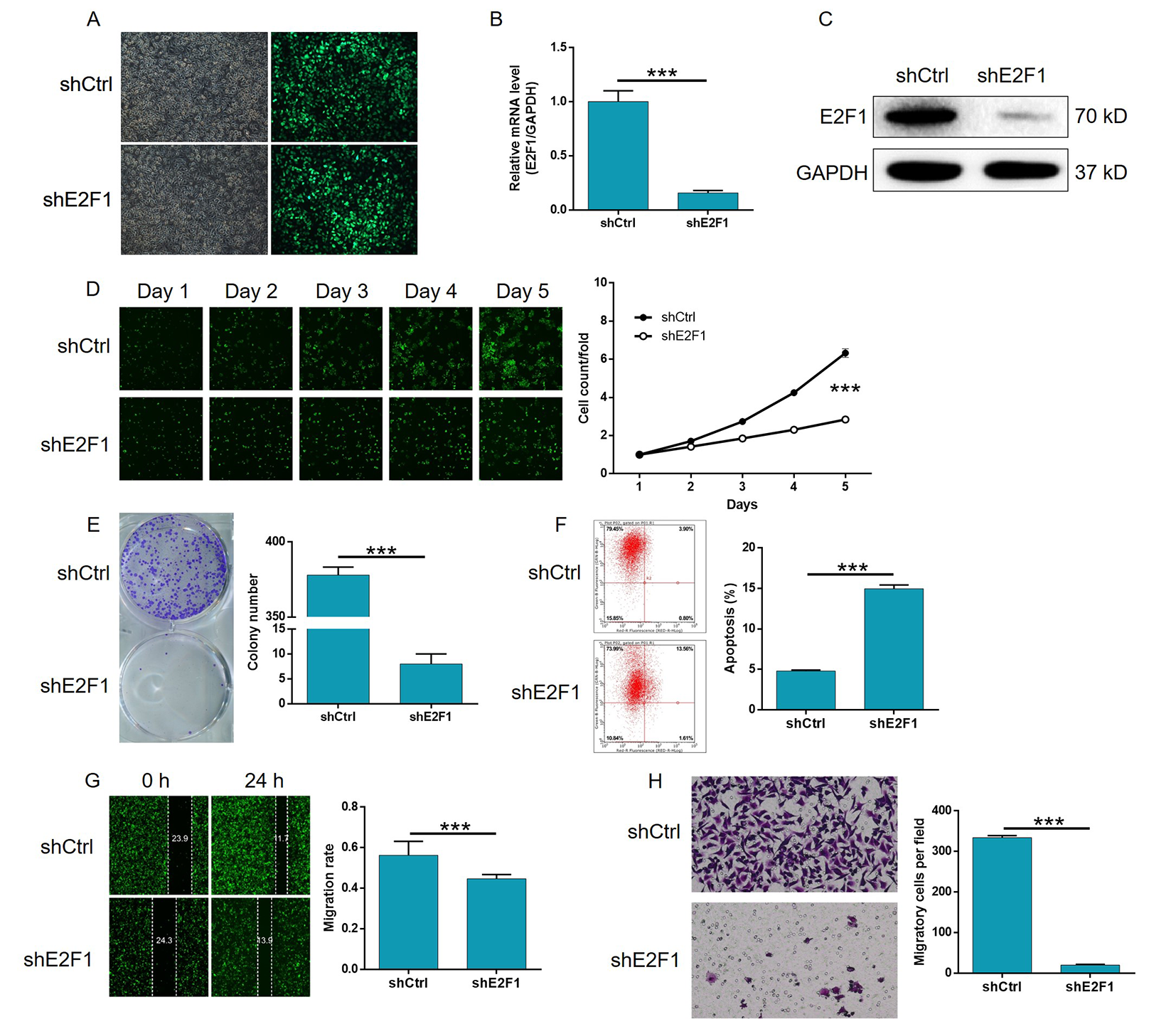

Supplement: Supplementary file 10 — Figure S6. [file 41419_2021_4148_MOESM10_ESM.tif]

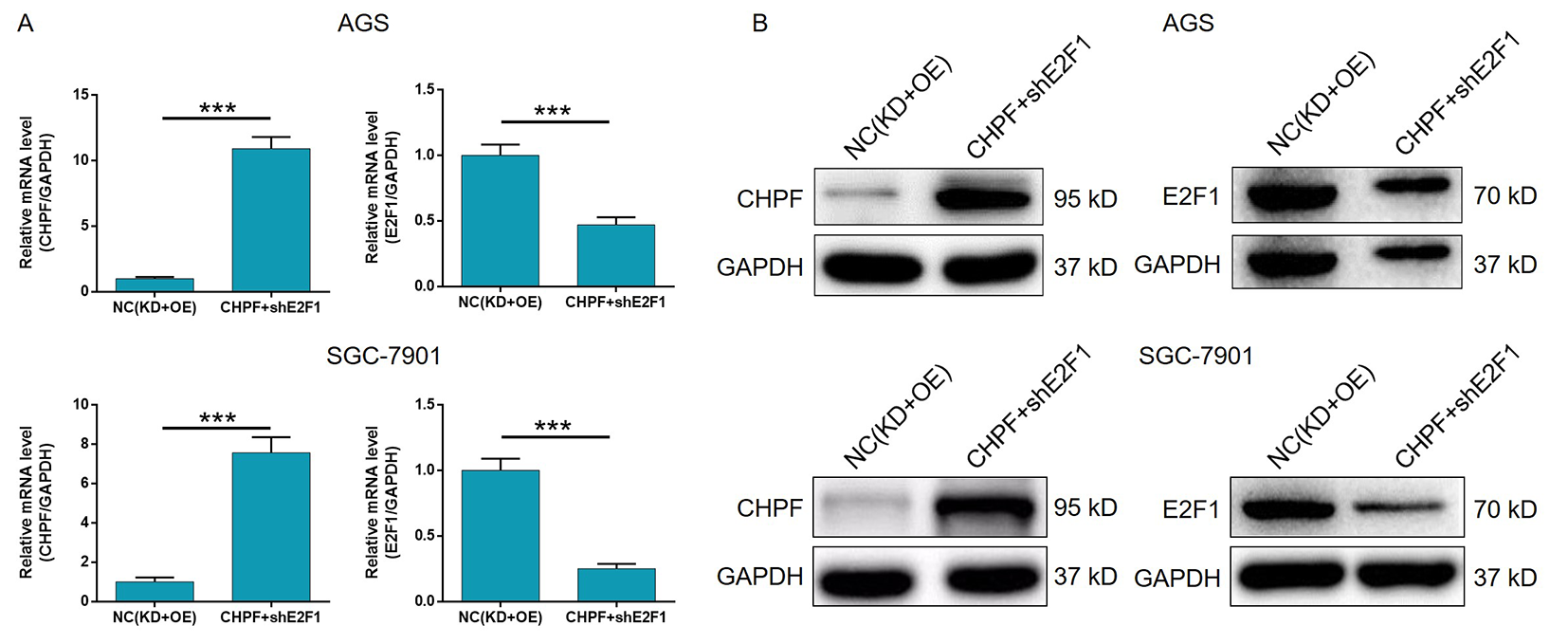

Supplement: Supplementary file 11 — Figure S7. [file 41419_2021_4148_MOESM11_ESM.tif]

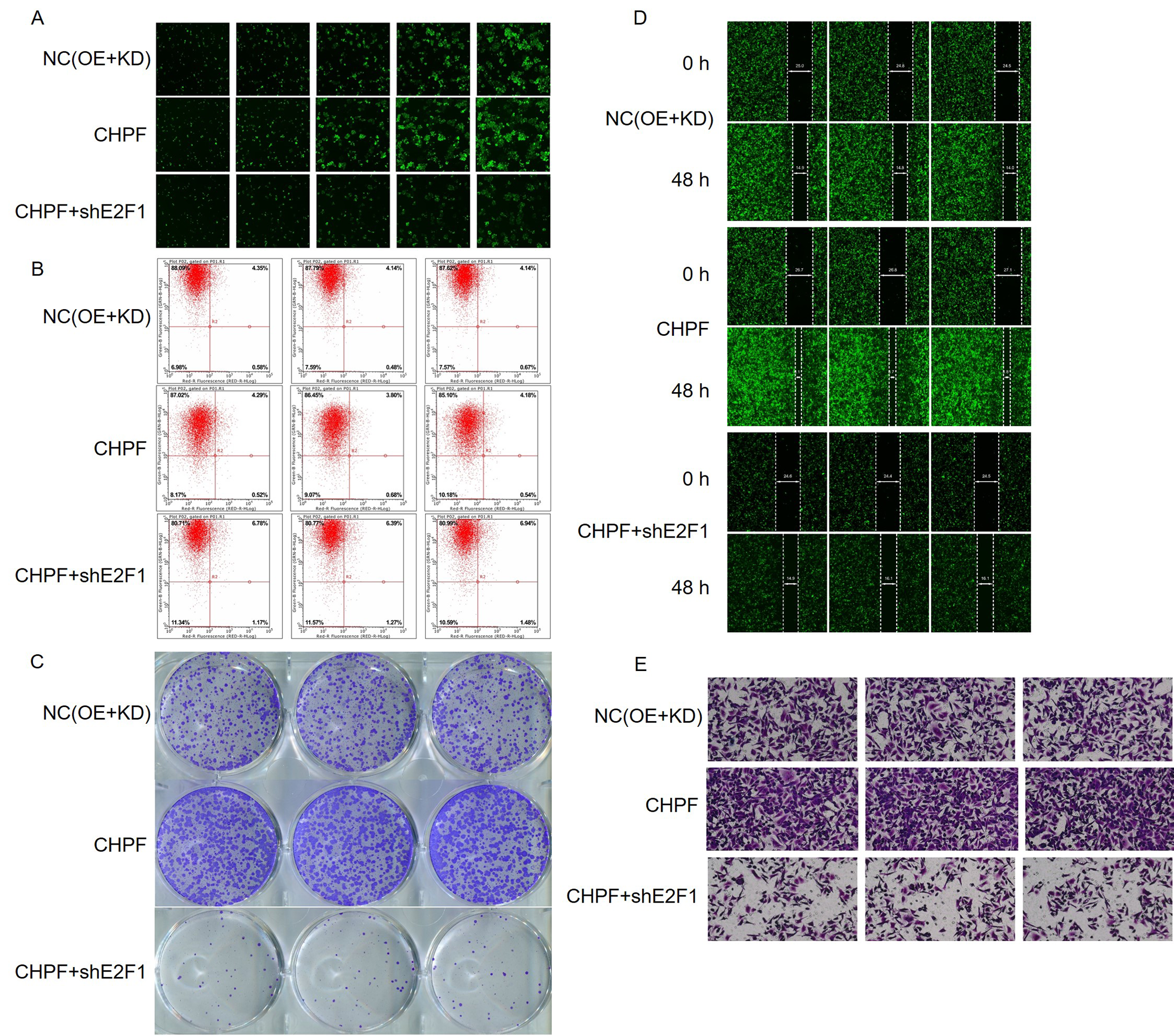

Supplement: Supplementary file 12 — Figure S8. [file 41419_2021_4148_MOESM12_ESM.tif]

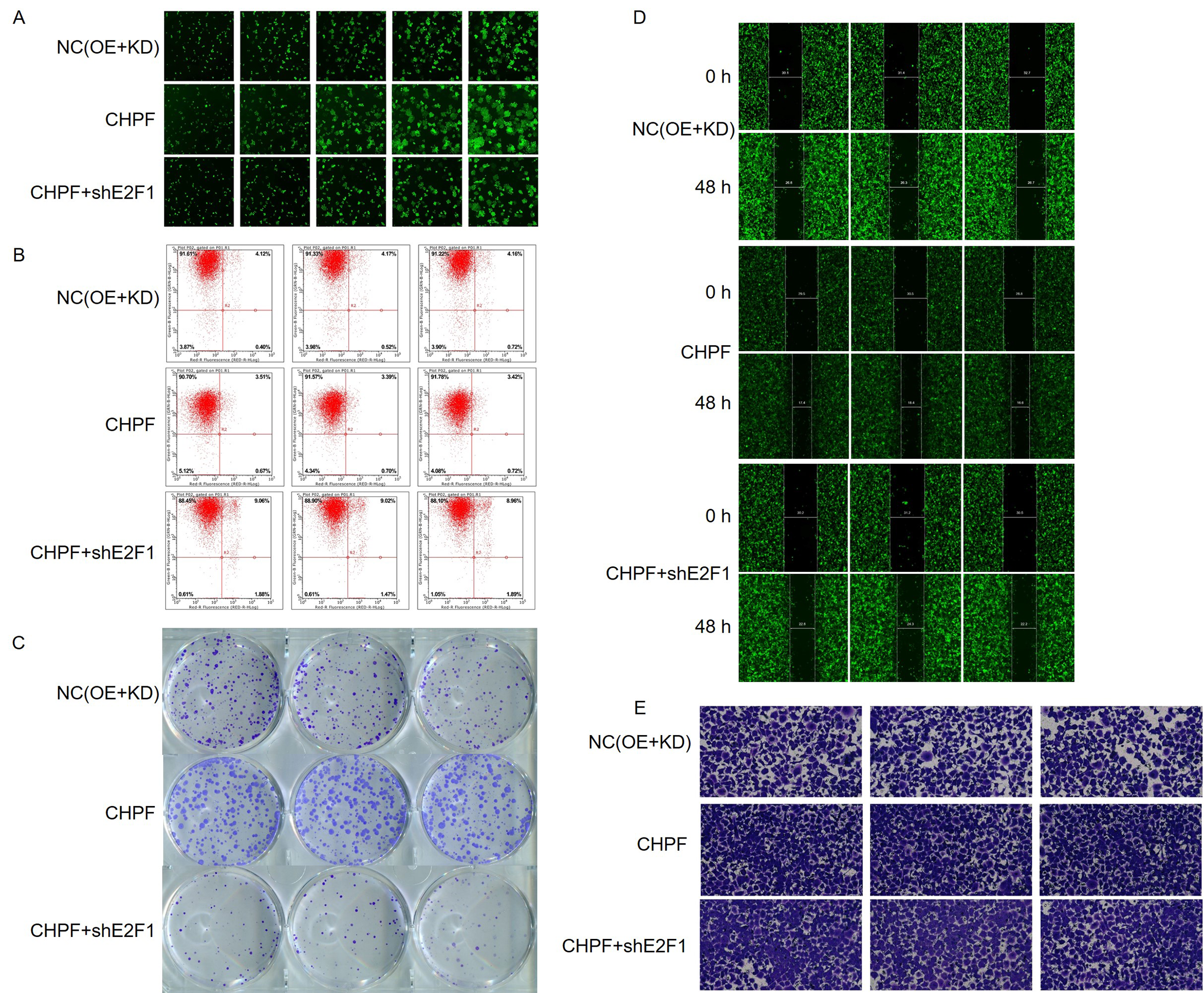

Supplement: Supplementary file 13 — Figure S9. [file 41419_2021_4148_MOESM13_ESM.tif]
